# Cascaded microsphere-coupled surface-enhanced Raman spectroscopy (CMS-SERS) for ultrasensitive trace-detection

**DOI:** 10.1515/nanoph-2021-0620

**Published:** 2022-01-04

**Authors:** Yanlin Mi, Yinzhou Yan, Mengyuan Wang, Lixue Yang, Jing He, Yijian Jiang

**Affiliations:** Faculty of Materials and Manufacturing, Beijing University of Technology, Beijing 100124, China; Key Laboratory of Trans-scale Laser Manufacturing Technology (Ministry of Education), Beijing 100124, China; Beijing Engineering Research Center of Laser Technology, Beijing University of Technology, Beijing 100124, China

**Keywords:** microsphere, optical microcavity, plasmonics, surface-enhanced Raman spectroscopy (SERS)

## Abstract

Surface-enhanced Raman spectroscopy (SERS) has been widely investigated and employed as a powerful optical analytical technique providing fingerprint vibrational information of molecules with high sensitivity and resolution. In addition to metallic nanostructure, dielectric micro-/nano-structures with extraordinary optical manipulation properties have demonstrated capability in enhanced Raman scattering with ultralow energy losses. Here we report a facile cascaded structure composed of a large microsphere (LMS) and a small microsphere array with Ag nanoparticles as a novel hybrid SERS substrate, for the first time. The cascaded microsphere-coupled SERS substrate provides a platform to increase the molecular concentration, boost the intensity of localized excitation light, and direct the far-field emission, for giant Raman enhancement. It demonstrates the maximum enhancement factor of Raman intensity greater than 10^8^ for the limit of detection down to 10^−11^ M of 4-nitrothiphenol molecules in aqueous solution. The present work inspires a novel strategy to fabricate cascaded dielectric/metallic micro-/nano-structures superior to traditional SERS substrates towards practical applications in cost-effective and ultrahigh-sensitive trace-detection.

## 1 Introduction

Raman spectroscopy, providing fingerprint vibrational information, is a powerful tool for identification of material and molecular species. However, the ultralow cross section of Raman scattering is the major obstacle towards practical applications. To overcome the drawback, several enhanced-Raman techniques have been developed, e.g. plasmon-enhanced-Raman scattering (PERS), interference-enhanced Raman scattering, resonance Raman scattering, and coherent anti-Stokes Raman scattering, etc. [1], [2], [3], [4]. The development of PERS techniques, including surface-enhanced Raman spectroscopy (SERS) [5, 6], tip-enhanced Raman spectroscopy [7, 8], shell-isolated nanoparticle-enhanced Raman spectroscopy [9, 10], etc. have been witnessed to facilitate spontaneous Raman scattering to applications for over past two decades. SERS technique has been widely explored for ultrasensitive detection of analytes in various fields, e.g. biochemical characterization, hazardous material inspection, food safety, etc. [11], [12], [13], [14], [15]. It can achieve ultrahigh enhancement factors of Raman intensity (*EFRI*) for single-molecule level detection due to the giant near-field electromagnetic (EM) and hot-electron-transfer-induced chemical enhancement [16, 17]. The near-field EM enhancement, originated from localized surface plasmon resonances (LSPRs) between adjacent metallic nanoparticles (*NP*s) for ‘hot spots’, has been well-acknowledged to be predominant to SERS [18]. A variety of SERS substrates were therefore synthesized with different metallic *NP*s (e.g. Au, Ag, and Cu, etc.) in colloids [19], [20], [21] or on rigid/flexible substrates [22], [23], [24], [25]. Unfortunately, the balance between performance, cost, reproducibility and stability cannot be well-weighted so far, limiting the SERS technique used in practical applications.

To overcome the inherent drawbacks of SERS, non-plasmon-enhanced Raman spectroscopies were developed. The non-plasmon-enhanced Raman spectroscopic substrates were composed of dielectric microstructures with tens of wavelengths [26, 27]. It suppresses the inherent ohmic energy losses in metallic/semiconducting nanostructures and confines the hot spots in the microstructure with high spatial controllability. The developed dielectric Raman enhancers, e.g. optical microcavities [28, 29], Mie antennas [30, 31], photonic crystals [32, 33], etc. were witnessed of the *EFRI* competitive to plasmonic enhancers. The dielectric microsphere, as a representative of non-plasmonic microcavity, was attracted considerable attention on Raman enhancement in the past decade [34], [35], [36], [37], [38]. The microsphere cavity supports several extraordinary optical phenomena, including Mie-induced photonic nanojet (PNJ) [39], optical whispering-gallery mode (WGM) [40], and directional antenna effect (DA) [41, 42]. The enhanced Raman detection via the use of bare dielectric microsphere array was first experimentally reported in 2007, for the *EFRI* of ∼10^4^ ascribing to the focusing property. Then, the microsphere-based Raman enhancement via the WGMs was investigated [43]. Until 2015, the contributions of PNJ, WGM and DA to microsphere-enhanced Raman spectroscopy (MERS) were theoretically revealed and experimentally validated [44]. The microsphere cavity demonstrated the compatibility to organic substrates achieving flexible MERS for improvement of *LoD* of crystal violet and Sudan I molecules by 1 orders of magnitude [45].

However, the *EFRI* of MERS without assistance of plasmonics (for ∼10^2^ folds) was far lower than the SERS with LSPR hot-spots (>10^5^ folds). The combination of MERS with SERS (MERS-SERS) for higher *ERRI* was therefore proposed by merging metallic nanostructures into microsphere cavities, cataloging WGM-enhanced and PNJ-enhanced LSPRs [46–51]. For WGM-enhanced LSPRs, Wang et al. synthesized silver-coated polystyrene microspheres for *LoD* of rhodamine 6G (R6G) molecules down to 10^−6^ M [47]. Yang et al. fabricated SiO_2_@Si core-shell arrays decorated with Au*NP*s for R6G down to 10^−10^ M [48]. The PNJ-enhanced LSPRs was first theoretically predicted in 2006, by which the *EFRI* via combining a microsphere with a Ag*NP* dimer was 10^13^ [49]. Until 2014, the experimental demonstration was performed, in which the *LoD* for methylene blue molecules was reduced by 5 orders of magnitude [50]. Gour et al. further theoretically demonstrated the capability of PNJ generated by microsphere cavity to couple LSPRs in nanoshell dimers, providing an extra *EFRI* of 10^3^ in addition to LSPRs [51]. The great difference of *EFRI*s between experiments and theories of microsphere-enhanced LSPRs should be attributed to the collimation of excitation light to near-field hot-spots, in which the efficient energy localization by the microsphere cavity focusing into *NP*s was critical. Therefore, understanding the excitation energy localization and developing a self-collimated hybrid Raman enhancer was of importance to realize ultrasensitive Raman trace-detection for the microsphere-coupled SERS substrates.

In this work, we proposed a novel cascaded microsphere-coupled SERS (CMS-SERS) enhancer, composed of a large microsphere (LMS), a small microsphere array (SMS) and Ag*NP*s. The hierarchical structure possessed self-collimated property for efficient energy localization and directional emission from the hybrid structure of LMS/SMS/Ag*NP*s for giant Raman enhancement. The contributions of PNJ, DA, and LSPR to Raman enhancement were revealed theoretically and confirmed experimentally, by which the structural parameters of CMS-SERS enhancer were optimized to achieve the ultrasensitive trace-detection. The present work not only opens up a facile strategy to design and fabricate hybrid Raman enhancers towards cost-effective spectroscopic detection, but also advances the optical manipulation in dielectric microcavity and metallic *NP*s, beneficial for development of novel hybrid nanophotonic devices with high efficiency in the future.

## 2 Experiment

### 2.1 Fabrication of cascaded microsphere-coupled SERS enhancers

**Figure 1:**
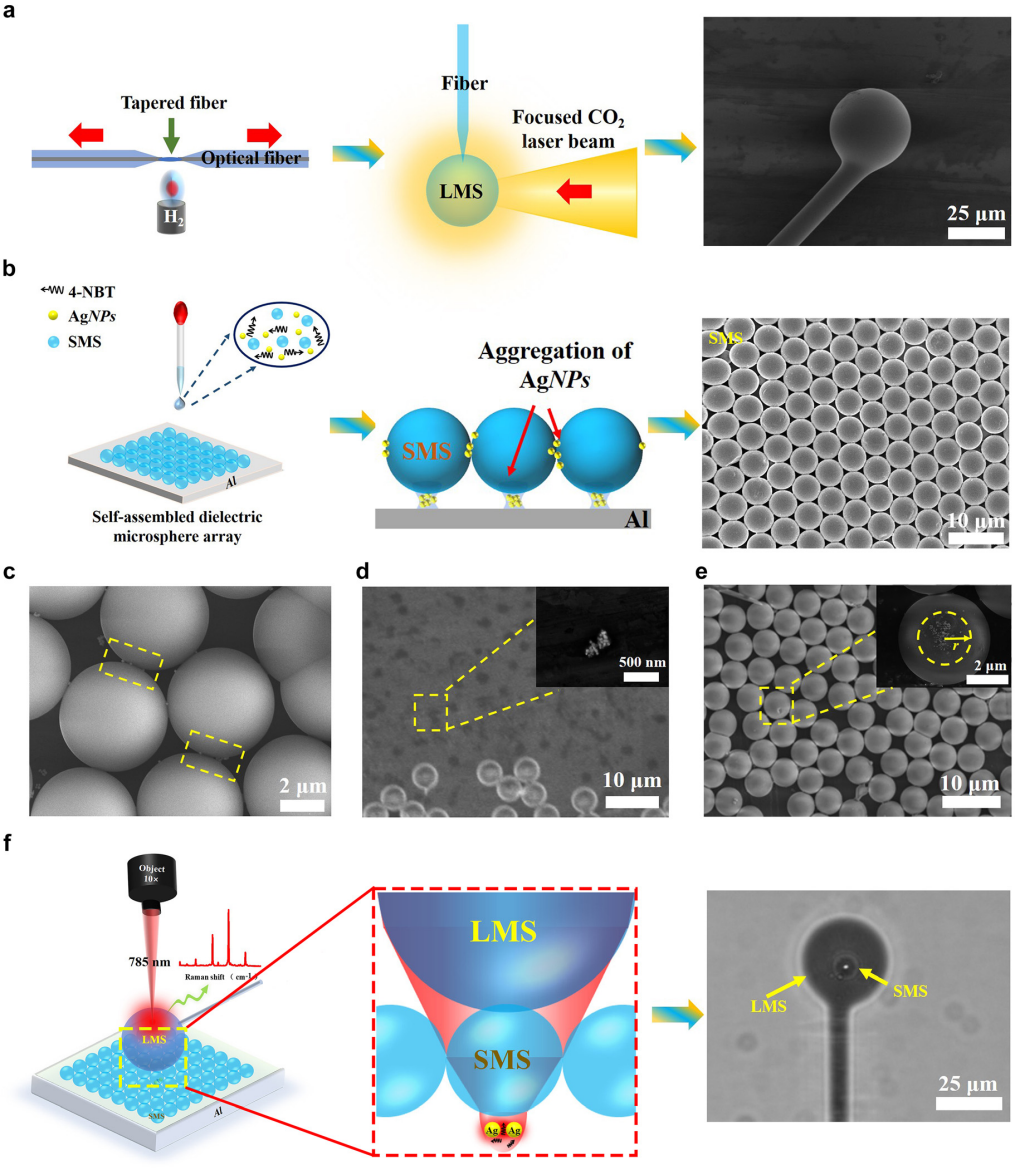
Schematic of CMS-SERS enhancer. (a) Fabrication of LMS via laser melting of tapered fibers. (b) Self-assembly of SMS with Ag*NP*s by drop-coating. (c)–(e) Close-up views of (c) gaps between SMS, (d) Ag*NP*s on Al substrate at the contact points of SMS, and (e) *AgNP*s aggregated on bottoms of SMS. (f) Typical structure of CMS-SERS enhancer, composed of LMS, SMS, and Ag*NP*s, for enhanced-Raman trace detection.

The structure of CMS-SERS enhancer is shown in Figure 1, composed of three parts, i.e. an LMS, an SMS, and Ag*NP*s. The LMS was first fabricated by following procedures. A commercial single-mode fused silica fiber (G652D, Changfei Co. Ltd., China) with a typical refractive index of 1.45 in the visible band was melted and tapered with a diameter in the range of 10–100 μm using the fiber fused biconical tapered system (FSCW-3000, Shandong Fushuoguang Electronic Communications Co. Ltd., China) with the hydrogen rate of 220 mL/min and drawing speed of 3000 μm/s, as shown in the left panel of Figure 1(a). Then, the tapered fiber was cut off and the head of tapered fiber was melted by a CO_2_ laser (FSTI60SFG, Synard, USA) using a laser power of 40–50 W with a focal spot of 2 mm, as demonstrated in the middle panel of Figure 1(a). The LMS was thereby formed on the head of tapered fiber due to the surface tension of molten silica, as illustrated in the right panel of Figure 1(a). The size of LMS was dependent upon the laser power as well as the diameter and length of tapered fiber fed into the laser-melting zone, by which the diameter of LMS was controllable from 20 μm to 200 μm.

The fused-silica microspheres with diameters of 4.86 ± 0.02 μm (Bang Laboratories Co., Ltd, USA) were employed to form the SMS. The colloidal solution of Ag*NP*s with the mean diameter of 30 nm (Perser Testing Certification CO., Ltd, China) was used as the SERS enhancers. The 4-nitrothiophenol (4-NBT) molecules (Beijing Huaweiruike Chemical Co. Ltd., China) were chosen as the Raman reporter. The SMS and Ag*NP*s were first mixed with the 4-NBT solution with concentration down to 10^−12^ M, depositing onto the aluminum substrate as shown in the left panel of Figure 1(b). A close-packed SMS monolayer was then self-assembled, in which the Ag*NP*s with 4-NBT molecules were aggregated to the bottoms and gaps of SMS, as shown in the middle panels of Figure 1(b). The right panel of Figure 1(b) further demonstrates the overview of close-packed SMS with Ag*NP*s and 4-NBT molecules. The close-up views of the gaps between SMS (as Figure 1(c)), the SMS contact points on Al substrate (as Figure 1(d)), and the bottoms of SMS (as Figure 1(e)) by SEM (SU-9000, Hitachi High-Tech Europe GmbH, German) further confirmed the aggregation of Ag*NP*s, indicating the increased concentration of 4-NBT molecules at these positions. Finally, the LMS was placed onto the SMS achieving the CMS-SERS structure, as shown in the right panel of Figure 1(f) via the optical microscope under the reflection mode (BX-51, Olympus Co. Ltd., Japan).

### 2.2 Acquisition of Raman spectra

The Raman spectra were acquired by a SmartRaman confocal-micro-Raman system (developed by Institute of Semiconductors, Chinese Academy of Sciences) with a 10×/*NA*0.25 objective (Olympus MPlan N) under the backscattering geometry. The collected Raman signals were analyzed by the Horiba LabRAM iHR550 spectrometer with a 600 lines/mm grating and a low-noise CCD detector. The excitation source was a 785 nm narrow-linewidth laser (MDL-E-785, Changchun New Industries Optoelectronics Technology Co., Ltd., China), of which the power arriving onto the sample surface was 300 μW with a spot size of 25 μm. The integration time for Raman detection was set to be 10 s. The fingerprinting range of Raman spectrum in 600–1800 cm^−1^ was acquired to estimate the *EFRI*s and *LoD*s of CMS-SERS enhancers. The *EFRI*s of different enhancers was calculated by [52].

(1) $$\mathrm{EFRI}=\frac{I_{ERS}}{I_{0}}\times \frac{C_{0}}{C_{ERS}}$$

where *I*_ERS_ and *I*_0_ are the intensities of enhanced and original Raman peaks, respectively; *C*_ERS_ and *C*_0_ are the corresponding concentrations of Raman reporter molecules in aqueous solutions. It should be noted that the *EFRI* used in this work was identified by concentration, rather than the number of molecules [53]. The aggregation of Raman reporter molecules with Ag*NP*s at the bottom of SMS during solution drying was considered to highlight the contribution of molecular aggregation to Raman enhancement. The enhancement factor by aggregation (*EFRI*_aggre_) was estimated by (*r*_SMS_/*r*)^2^, where *r* is the radius of aggregation region after solution drying and *r*_SMS_ is the radius of SMS, by which the *EFRI*_aggre_ was calculated to be 2.7 according to the evaluation shown in the inset of Figure 1(e).

### 2.3 Numerical simulation

The numerical simulation of EM field was performed in Comsol Multiphysics software package (licensed by Comsol Co., Ltd.). A 2D cross-sectional model was developed to calculate the electric field in the CMS-SERS structures. The LSPR between Ag*NP*s as well as the PNJ and DA by LMS/SMS for optical regulation were numerically simulated. The diameters of LMS and SMS for numerical analysis were in the rage of 20–60 μm and 1–10 μm, respectively. The refractive indexes of LMS and SMS were both set as 1.45. The nanogaps of Ag*NP*s was fixed at 1.2 nm according to the SEM evaluation. The complex permittivity of Ag*NP*s was set as −29.789 + 0.376i at 785 nm. Perfectly matched layers were applied as the boundary conditions. The 785 nm planewave was used to simulate PNJ and LSPR for excitation, whereas the electric dipoles with the same wavelength were employed to study the manipulation of DA and LSPR to far-field scattering.

## 3 Results and discussion

### 3.1 Configuration and sensitivity of CMS-SERS enhancers for Raman tract-detection

**Figure 2:**
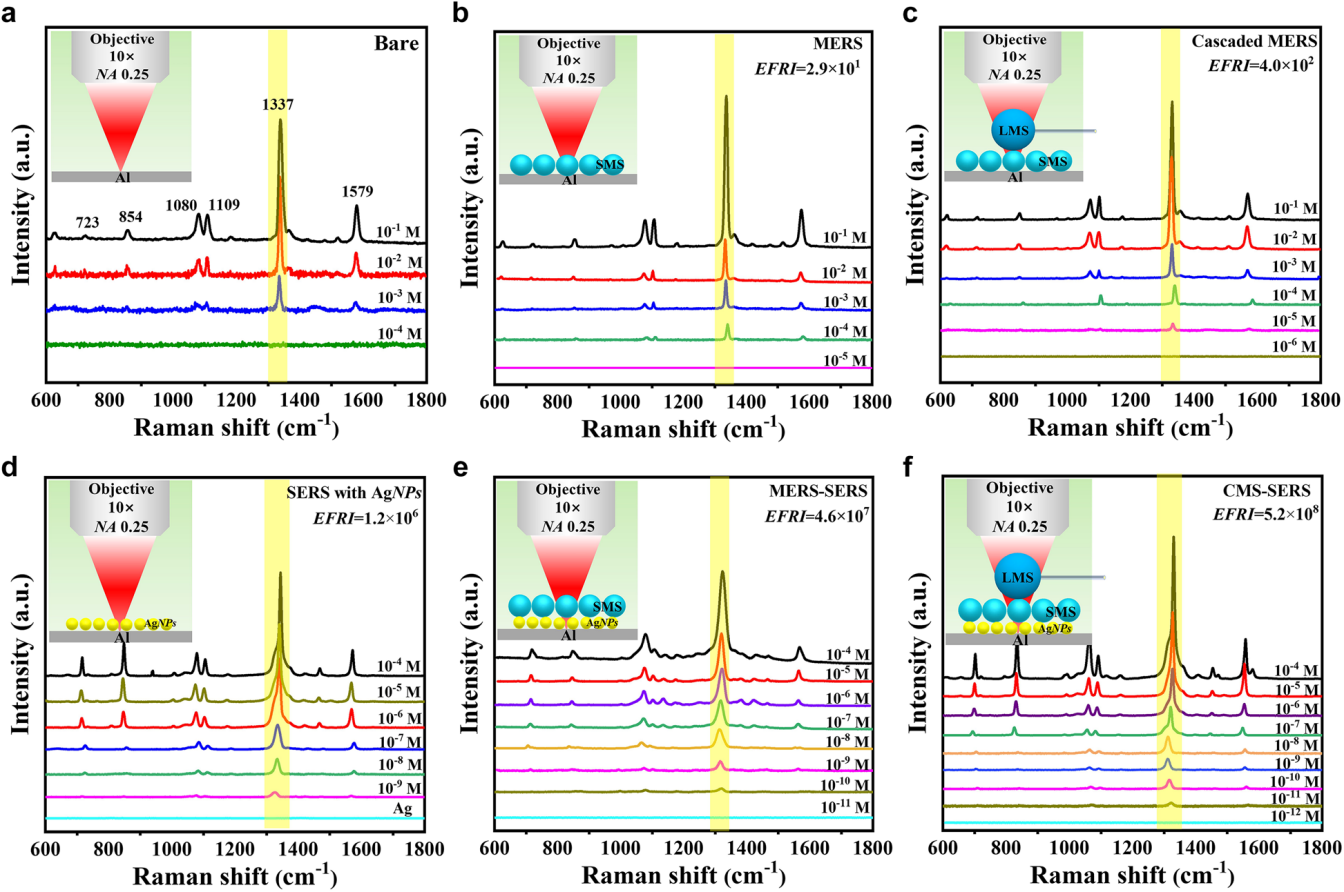
Evaluation of enhancement factor and sensitivity of Raman scattering by various configurations with (a) bare Al substrate, (b) MERS enhancer (with SMS), (c) cascaded MERS enhancer (with LMS/SMS), (d) SERS enhancer (with Ag*NP*s), (e) MERS-SERS enhancer with (SMS/Ag*NP*s), and (f) CMS-SERS enhancer with a LMS/SMS/Ag*NP*s structure, for 4-NBT down to 10^−11^ M in aqueous solution.

The sensitivity of CMS-SERS enhancers was investigated in Figure 2, where the bare, MERS (with SMS), cascaded MERS (with LMS/SMS), SERS (with Ag*NP*s), and MERS-SERS (with SMS/Ag*NP*s) substrates were used as the controls. Figure 2(a) illustrates the characteristic Raman bands of 4-NBT solution with a high concentration of 10^−3^ M, including the dominant modes of *π*(CH) + *π*(CS) + *π*(CC) @ 723 cm^−1^, *π*(CH) @ 854 cm^−1^, *ν*(CS) @ 1080 cm^−1^, *ν*(CH) @ 1109 cm^−1^, *ν*(NO_2_) @ 1337 cm^−1^, and *ν*(CC) @ 1579 cm^−1^ [54], [55], [56], [57], [58]. The peak of *ν*(NO_2_) @ 1337 cm^−1^ with the maximum Raman intensity was selected to identify the *EFRI*s and *LoD*s of 4-NBT. According our pilot experiments, the diameters of LMS and SMS were optimized for the maximum *EFRI*s, i.e. 4.86 μm and 36 μm, respectively. Figure 2(b) shows the Raman enhancement by MERS, in which *ERFI*_MERS_ = 2.9 × 10^1^ for the *LoD* reduced by 1 order of magnitude down to 10^−4^ M. The cascaded MERS substrate by covering LMS onto SMS further increased *ERFI*_cas-MERS_ by 1 order of magnitude to 4.0 × 10^2^, by which the *LoD* of 10^−5^ M was achieved as Figure 2(c). Figure 2(d) shows the Raman spectra enhanced by the SERS substrate with Ag*NP*s, demonstrating a high *EFRI*_SERS_ up to 1.2 × 10^6^ with the *LoD* of 10^−9^ M due to the LSPRs for near-field electric enhancement and hot electron transfers for chemical enhancement [59]. The enhancement strength was in good agreement with the literatures using Ag*NP* colloids [60], [61], [62], [63]. The coupling of MERS with SERS, as Figure 2(e), illustrated a dual enhancement with *EFRI*_MERS-SERS_ = 4.6 × 10^7^ further decreasing the *LoD* for 10^−10^ M. Then the LMS covered onto the SMS with Ag*NP*s exhibited the maximum *EFRI*_CMS-SERS_ up to 5.2 × 10^8^, by which the *LoD* of 10^−11^ M was realized by the hierarchical structure composed of dielectric cascaded microspheres and Ag*NP*s, as shown in Figure 2(f). It can be clearly seen that the presence of cascaded microspheres provided an efficient enhancement channel for 2 orders of magnitude in addition to LSPRs in Ag*NP*s facilitating ultrasensitive Raman sensing.

### 3.2 Photonic nanojets and parameter optimization of CMS-SERS enhancers

**Figure 3:**
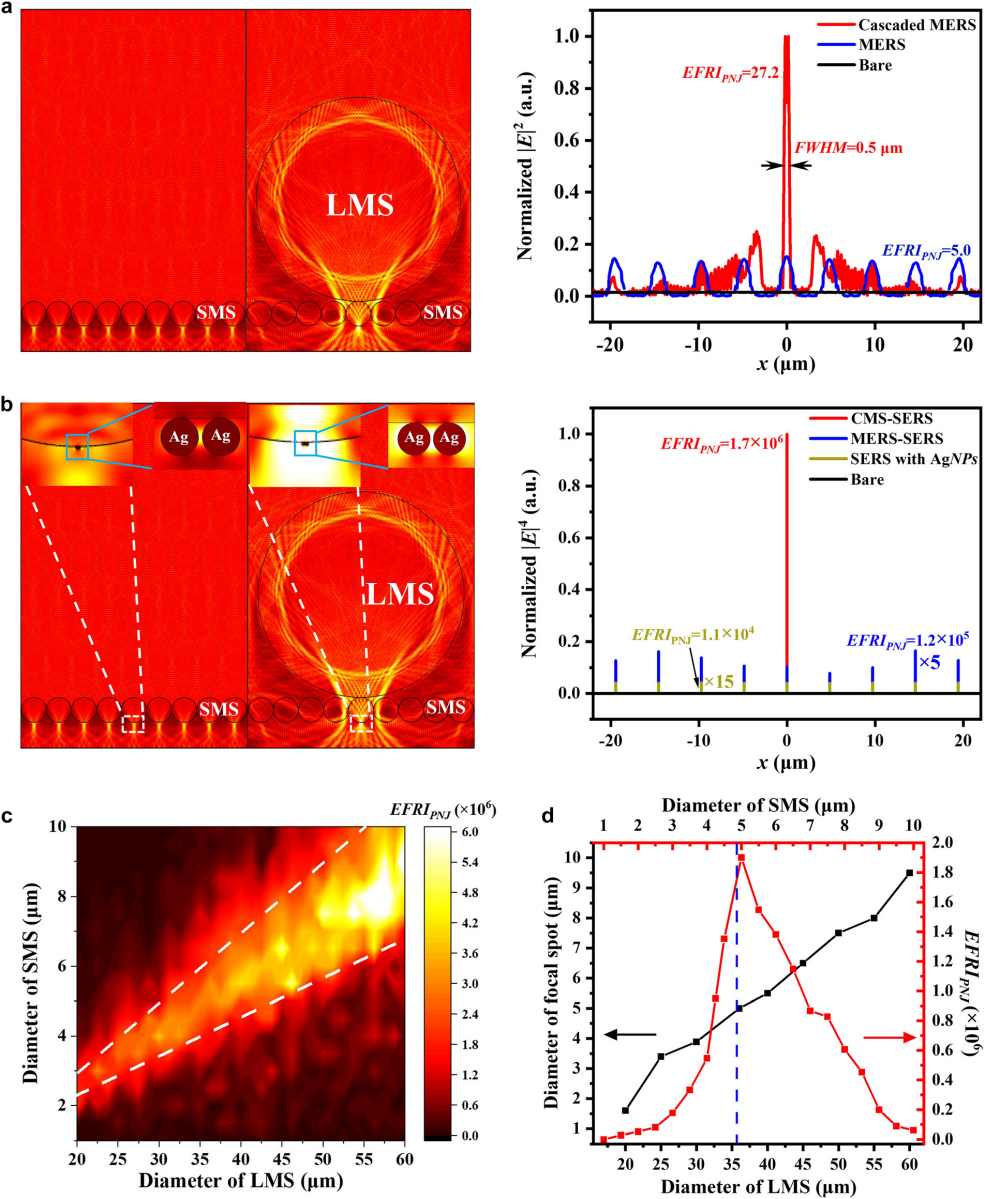
Numerical simulation of PNJs generated by CMS-SERS enhancers with different configurations. (a) Intensity distribution of electric fields focused by MERS and cascaded MERS, and the corresponding *EFRI*_PNJ_. (b) Intensity distribution of electric fields focused by MERS-SERS and CMS-SERS, and the corresponding *EFRI*_PNJ_. (c) Evolution of *EFRI*_PNJ_ with diameters of LMS and SMS. (d) Evolutions of focal spot size with LMS diameter and extracted *EFRI*_PNJ_ with diameter of SMS covered by a 36 μm-diameter LMS.

The PNJ generated by microsphere cavity contributed to the excitation efficiency for Raman enhancement [43]. The concentration of 4-NBT to the bottoms of SMS as mentioned in Figure 1(d) and (e) indicated that the *EFRI* via PNJ (*EFRI*_PNJ_) was linearly dependent upon the PNJ intensity, i.e. |*E*_exc_|^2^ . Figure 3(a) shows the focusing properties of SMS and cascaded LMS/SMS structures, respectively. It can be seen that the 4.86 μm-diameter SMS generated a series of PNJs, of which the spot sizes are ∼1.2 μm at the bottom side of SMS. When a 36 μm-diameter LMS covered on the SMS, the LMS focused the excitation laser collimating into a small microsphere and therefore dramatically enhanced the PNJ intensity at the bottom of SMS. The cascaded MERS structure confined the excitation energy into a PNJ, of which the diameter was reduced to 0.5 μm close to the diffraction limit of 785-nm laser as shown in Figure 3(a). The corresponding *EFRI* by PNJ (*EFRI*_PNJ_) under MERS and cascaded MERS enhancers were therefore identified to be 5.0 and 27.2, respectively, with respect to the excitation intensity without microspheres.

Figure 3(b) shows the excitation intensity distributions of MERS-SERS and CMS-SERS enhancers via coupling of Ag*NP*s. The PNJs generated from the SMS and LMS/SMS boosted the LSPRs in the gaps between Ag*NP*s. It was well-acknowledged that the *EFRI* by LSPRs followed the |*E*|^4^ approximation for assumption of zero-Stokes shifts [34]. Therefore, the distributions of |*E*|^4^ were calculated, as shown in the right panel of Figure 3(b). It can be seen that the electric intensities of EM fields in the gaps between Ag*NP*s were significantly boosted, where the *EFRI* by SERS (*EFRI*_S*ER*S_) were 1.1 × 10^4^. It should be noted that the 2 orders of magnitude lower than the experimental value (1.2 × 10^6^) was attributed to the chemical enhancement (*EFRI*_chem_ = 1.0 × 10^2^) owing to the hot electron transfer from Ag*NP*s to 4-NBT molecules [64]. The corresponding PNJ-induced *ERFI*_PNJ_ were calculated to be 1.2 × 10^5^ and 1.7 × 10^6^, respectively, for MERS-SERS and CMS-SERS configurations. The coupling of SMS and LMS/SMS with aggregated Ag*NP*s resulted in the *EFRI* further increased by 29- and 417-fold, showing good agreement with the experimental results (38- and 433-fold) as estimated from Figure 2(d)–(f). The contribution of PNJs focused by LMS/SMS was confirmed to regulate the Raman enhancement by LSPRs, where the strong focusing strength was beneficial to the LSPRs for improvement of Raman enhancement.

To optimize the structural parameters of CMS-SERS enhancers, the effect of diameters of LMS and SMS on *EFRI*_*PNJ*_ was systemically studied, as Figure 3(c), with the diameter ranges of LMS and SMS in 20–60 μm and 1–10 μm, respectively. It can be found that there existed an optimal SMS diameter with respect to a specific LMS diameter for the maximum *EFRI*_PNJ_. Moreover, the maximum *EFRI*_PNJ_ was increased with the diameters of SMS and LMS increasing simultaneously. To further reveal the match strategy for SMS and LMS diameters, the spot size focused by LMS with diameters of 20–60 μm was calculated. It can be seen that the spot size at the bottom of LMS was increased with the LMS diameter increasing, as shown in Figure 3(d). The evolution of *EFRI*_PNJ_ with SMS diameter at 36 μm-diameter LMS was then extracted from Figure 3(c) and plotted as the red line in Figure 3(d). It can be clearly seen that the maximum *EFRI*_PNJ_ appeared when the spot size (∼5 μm) focused by LMS was close to the diameter of SMS (∼4.86 μm). Therefore, the match between diameter of SMS and focal spot of LMS was validated to confine the most excitation energy into the gaps between Ag*NP*s at the bottom of SMS achieving highly-efficient LSPRs for Raman enhancement.

### 3.3 Directional antenna effect of CMS-SERS enhancers

**Figure 4:**
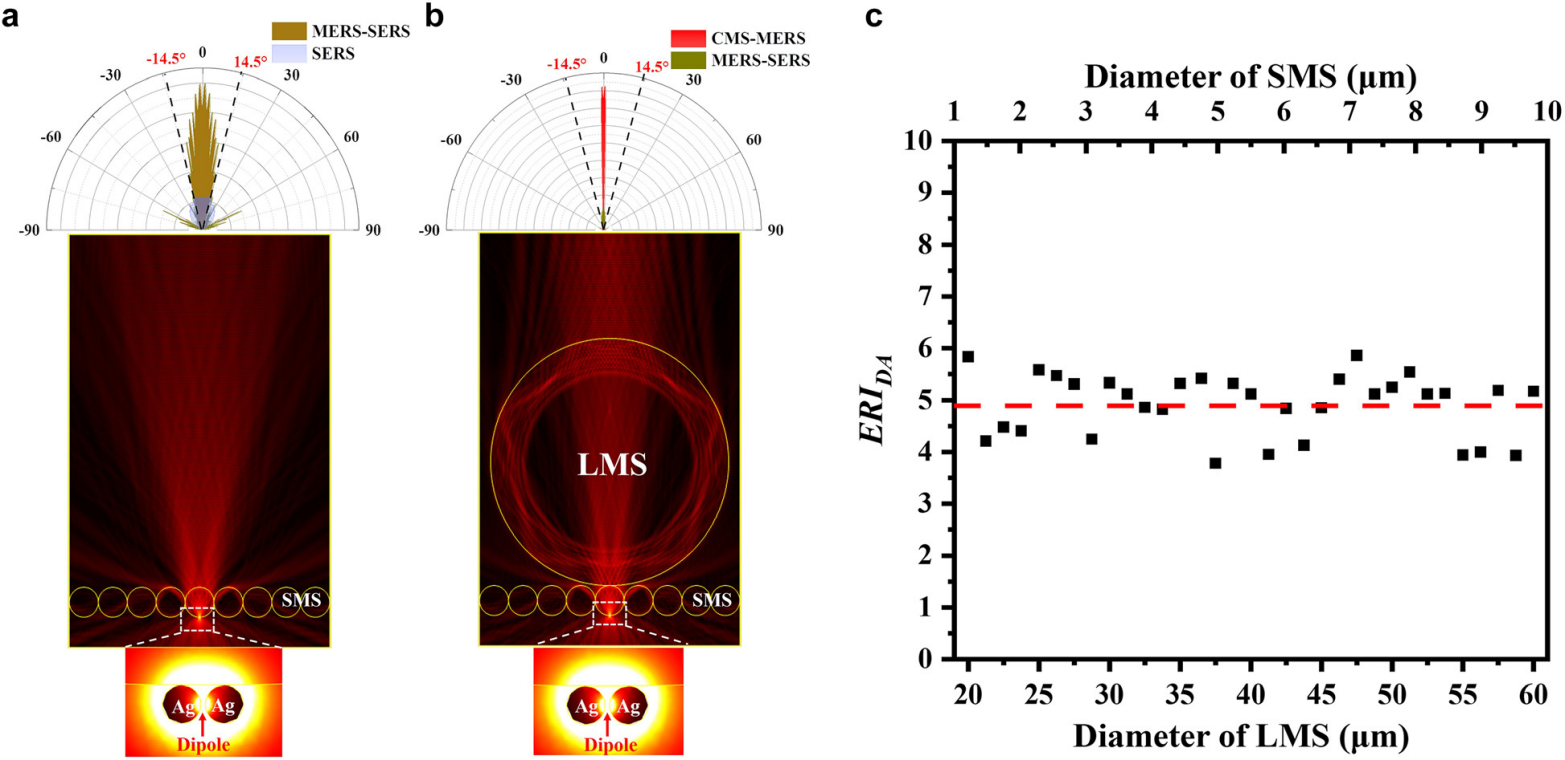
Numerical simulation of directional antenna effect and far-field emission patterns from (a) MERS-SERS and (b) CMS-SERS enhancers, where the black dash lines indicate the *NA* of objective. (c) Evolution of *EFRI*_DA_ with optimal diameter configuration of cascaded LMS/SMS.

The DA of microsphere cavity was the other channel for Raman enhancement in addition to PNJs. Figure 4 illustrates the intensity distributions of electrical fields from the MERS-SERS and CMS-SERS enhancers, respectively. It can be seen from the far-field emission polar pattern in Figure 4(a) that the directional emission was enhanced by the MERS-SERS structure. The CMS-SERS enhancer can further improve the isotropic emission along the optical axis through the LMS, SMS, and gap between Ag*NP*s for highly-directional EM confinement within a narrow divergence (<±1.2°) by self-collimation, as shown in Figure 4(b), beneficial for efficient collection of scattered light. For the objective with *NA*0.25, the *EFRI* via DA (*EFRI*_DA_) can be estimated by

(2) $$\mathrm{EFR}I_{DA}=\frac{{\int_{0}^{\sin^{-1}\left(NA\right)}\left|E_{DA}\left(\theta \right)\right|}^{2}\;\sin\;\theta d\theta }{{\int_{0}^{\sin^{-1}\left(NA\right)}\left|E_{SERS}\left(\theta \right)\right|}^{2}\;\sin\;\theta d\theta }$$

where *E*_DA_(*θ*) is the angular vector of electric field by MERS-SERS or CMS-SERS enhancer in the far field; *E*_SERS_(*θ*) is the angular vector of electric field by SERS in the far field. The calculated *EFRI*_DA_ were 3.9 and 5.4 for MERS-SERS and CMS-SERS structures, respectively, with respect to the SERS substrate, under the optimal configuration for the maximum *EFRI*_PNJ_, i.e. 4.86 μm-diameter SMS and 36 μm-diameter LMS. The contribution of DA to Raman enhancement were significantly lower than that of PNJ as mentioned above. Figure 4(c) illustrates the effect of cascaded LMS/SMS with the optimal diameter configuration (i.e. the spot size focused by LMS equal to the SMS diameter) on directional capability, where the *EFRI*_DA_ were insensitive to the structure parameters and fixed at 4.9 ± 0.6. As a result, the optimization for the highest PNJ intensity was predominant to boost Raman scattering in the CMS-SERS enhancers, whereas the DA provided a stable enhancement in the far field by the self-collimation from the LMS/SMS/Ag*NP*s structures.

### 3.4 Effect of Ag*NP* location on Raman enhancement in CMS-SERS enhancers

**Figure 5:**
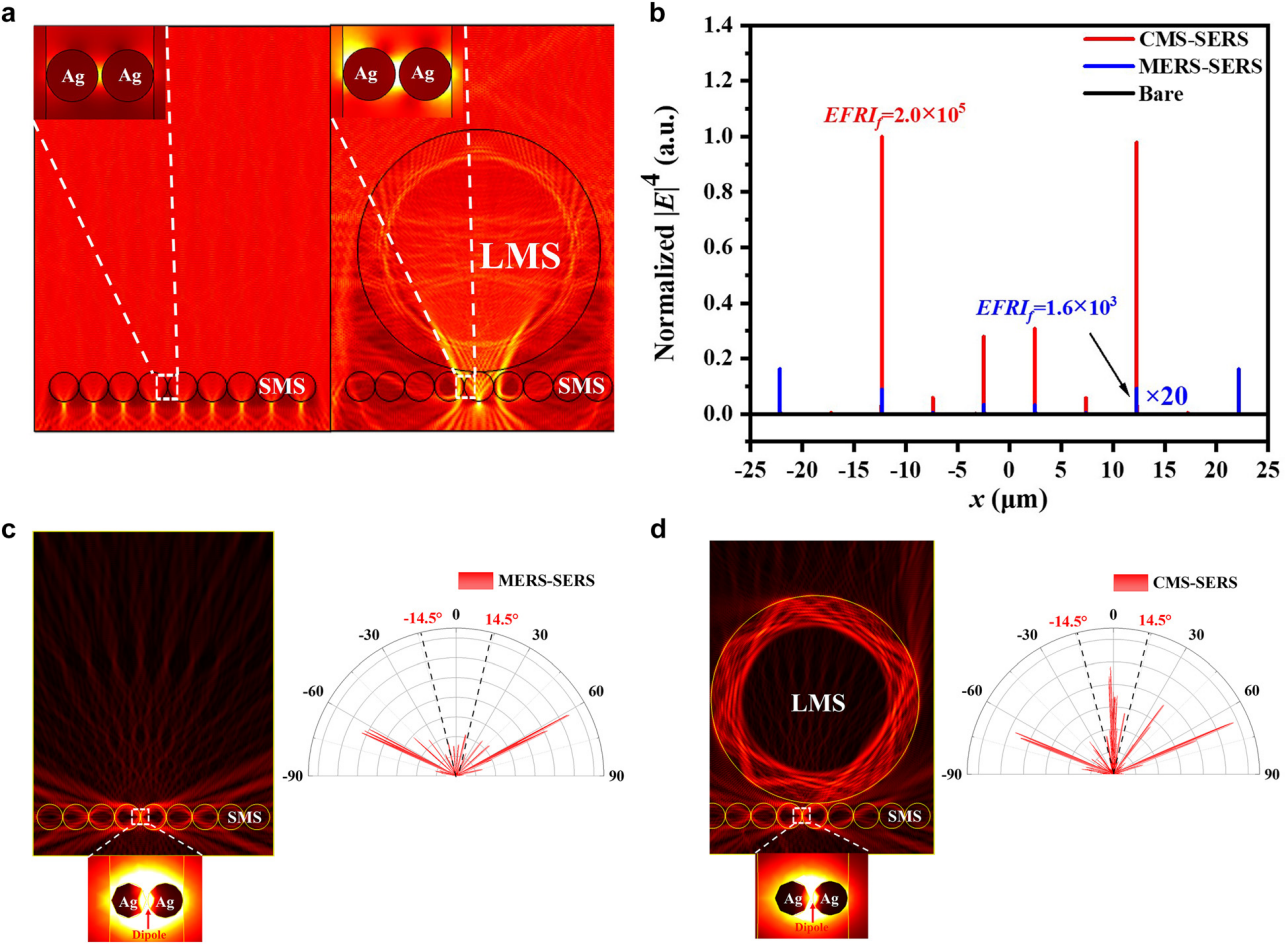
Numerical simulation of focusing property and directional antenna effect of CMS-SERS enhancers for Ag*NP*s located in the gaps between SMS. (a) Intensity distributions of electric fields in MERS-SERS and CMS-SERS configurations. (b) The corresponding *EFRI*_*f*_ for Ag*NP*s located in the gaps between SMS. (c) and (d) Electric fields and far-field emission patterns from (c) MERS-SERS and (d) CMS-SERS enhancers with the gap-attached Ag*NP*s.

In addition to the bottoms of SMS, the Ag*NP*s were also aggregated to the gaps between SMS as shown in Figure 1(c), i.e. gap-attached Ag*NP*s. The electric fields of excitation laser irradiating on these Ag*NP*s under MERS-SERS and CMS-SERS setups were numerically simulated as Figure 5(a). The intensity distributions of electric fields under different setups shown in Figure 5(b) further confirmed the negative contribution of SMS for gap-attached Ag*NP*s, where the *ERFI*_*f*_ estimated by the |*E*|^4^ approximation was 1.6 × 10^3^ for MERS-SERS, lower than SERS (with Ag*NP*s only) for 1 order of magnitude. This was attributed to the coupling of near-field energy from Ag*NP*s into the SMS, reducing the directional antenna effect of LSPRs on far-field emission. The LMS contributed to the majority of *EFRI*_*f*_, i.e. 2.0 × 10^5^ as illustrated in Figure 5(b). The effect of LMS in MERS-SERS and CMS-SERS enhancers on polar emission in the far field was also calculated as Figure 5(c) and (d). It can be seen that the SMS confined the emission light along the horizontal direction, dramatically reducing the *EFRI*_DA_ to 0.073. When the LMS covered onto the gap-attached Ag*NP*s, the capability of DA to collect and confine the horizontal emission within the objective was demonstrated, by which *EFRI*_*DA*_ was improved to 0.136. As a result, the *EFRI*_*f*_ × *ERFI*_DA_ in CMS-SERS enhancers was 2.7 × 10^4^. Although the *EFRI* was far lower than the Ag*NP*s aggregated to the bottoms of SMS (*EFRI*_PNJ_ × *ERFI*_DA_ × *ERFI*_aggre_ = 2.5 × 10^7^), the obvious enhancement for 2.7 × 10^6^ (considering the contribution of chemical enhancement ∼1.0 × 10^2^) still demonstrated the high tolerance of cascaded LMS/SMS structures to Ag*NP*s locations in practical applications.

### 3.5 Theoretical prediction and experimental validation for *EFRI*s of CMS-SERS enhancers

According to theoretical study on the contributions of PNJ and DA generated by microsphere cavity to Raman enhancement, the total *EFRI*s for MERS, cascaded MERS, SERS, MERS-SERS, and CMS-SERS enhancers were predicted as following

(3) $$EFRI=ERFI_{PNJ}\times EFRI_{chem}\times ERFI_{DA}\times ERFI_{aggre}$$

where the *EFRI*_PNJ_ includes the enhancement from microsphere focusing and LSPRs following the |*E*|^4^ approximation, and *EFRI*_*DA*_ represents the enhancement from direction antenna effect of SMS or cascaded LMS/SMS except LSPRs in Ag*NP*s. The calculated *EFRI*s by Eq. (3) for MERS, cascaded MERS, SERS, MERS-SERS, and CMS-SERS enhancers are listed in Table 1. It can be seen that the theoretically predicted values were in consistence with the experimental results, as listed in Figure 2. The maximum *EFRI* greater than 10^8^ for CMS-SERS realized the ultrasensitive detection for the *LoD* down to ∼10^−11^ M. It should be noted that the PNJ generated from the microsphere cavity contributed to the majority of Raman enhancement. The difference from theoretical calculation to experimental acquisition was ascribed to the aggregation of Ag*NP*s in gaps between SMS, resulting in the experimentally-measured *ERFI*s lower than theoretically-calculated ones. In this work, the cascaded MERS structure with an LMS covered onto SMS significantly reduced the quality factors of LMS and SMS, by which the WGMs was not observed from the CMS-SERS enhancer. Therefore, the contribution of WGMs to Raman enhancement was ignored.

**Table 1:** Theoretical calculations of *EFRI* for various Raman enhancers.

Enhancer	MERS	Cascaded MERS	SERS	MERS-SERS	CMS-SERS
*EFRI*_PNJ_	5.0	27.2	1.1 × 10^4^	1.2 × 10^5^	1.7 × 10^6^
*EFRI*_chem_	—	—	1.0 × 10^2^ (from Ag*NP*s)	1.0 × 10^2^ (from Ag*NP*s)	1.0 × 10^2^ (from Ag*NP*s)
*EFRI*_*DA*_	3.9	5.4	—	3.9	5.4
*EFRI*_aggre_	2.7	2.7	—	2.7	2.7
*EFRI*	52.6	396.6	1.1 × 10^6^	1.3 × 10^8^	2.5 × 10^9^

## 4 Conclusions

In conclusion, we constructed a facile CMS-SERS enhancer, composed of a large 36 μm-diameter LMS and a small 4.86 μm-diameter SMS with Ag*NP*s for ultrasensitive Raman detection, for the first time. The PNJ by cascaded microsphere cavities contributed to the majority of Raman enhancement by coupling with the LSPRs in gaps between Ag*NP*s. The *ERFI* up to 5.2 × 10^8^ was achieved for the *LoD* down to ∼10^−11^ M of 4-NBT in aqueous solution. In addition, the CMS-SERS enhancer demonstrated high tolerance to the locations of Ag*NP*s to reduce the requirement for collimation for the practical use. The theoretical prediction by the developed numerical model in good agreement with the experimental results further validated the Raman enhancement channels of the CMS-SERS enhancers from the increased molecular concentration, boosted intensity of localized excitation light, and directed far-field emission. The present work inspires a novel strategy to design and fabricate hybrid cascaded enhancers with dielectric/metallic micro-/nano-structures superior to traditional SERS substrates towards future applications in cost-effective and ultrahigh-sensitive spectroscopic trace-detection.

## Supporting Information

The supporting information is available from the corresponding author.

## Glossary

CMS-SERS: Cascaded microsphere-coupled surface-enhanced Raman spectroscopy
DA: Directional antenna effect
*EFRI*
: Enhancement factor of Raman intensity
EM: Electromagnetic
LMS: Large microsphere
*LoD*
: limit of detection
LSPR: Localized surface plasmon resonance
MERS: Microsphere-enhanced Raman spectroscopy
MERS-SERS: Combination of MERS with SERS
*NP*
: Nanoparticle
PERS: Plasmon-enhanced-Raman scattering
PNJ: Mie-induced photonic nanojet
R6G: Rhodamine 6G
SERS: Surface-enhanced Raman spectroscopy
SMS: Small microsphere array
WGM: Optical whispering-gallery mode
